# Case Report: Treatment of Kratom Use Disorder With a Classical Tricyclic Antidepressant

**DOI:** 10.3389/fpsyt.2021.640218

**Published:** 2021-03-31

**Authors:** Alessandro E. Vento, Simone de Persis, Sergio De Filippis, Fabrizio Schifano, Flavia Napoletano, John M. Corkery, Georgios D. Kotzalidis

**Affiliations:** ^1^ASL (Azienda Sanitaria Locale) Roma 2, Rome, Italy; ^2^Addictions' Observatory (ODDPSS), Rome, Italy; ^3^ASL, Rieti, Italy; ^4^Villa von Siebenthal Neuropsychiatric Clinic and Hospital, Genzano di Roma, Italy; ^5^Psychopharmacology, Drug Misuse & Novel Psychoactive Substances Research Unit, School of Life and Medical Sciences, University of Hertfordshire, Hatfield, United Kingdom; ^6^NESMOS Department (Neurosciences, Mental Health, and Sensory Organs), School of Medicine and Psychology, Sant'Andrea Hospital, Sapienza University, Rome, Italy

**Keywords:** kratom, mitragynine, substance use disorder, clomipramine, withdrawal syndrome

## Abstract

Kratom or *Mitragyna speciosa* (Korth.) is an evergreen tree of the coffee family native to South-East Asia and Australasia. It is used by locals recreationally to induce stimulant and sedative effects and medically to soothe pain and opiate withdrawal. Its leaves are smoked, chewed, or infused, or ground to yield powders or extracts for use as liquids. It contains more than 40 alkaloids; among these, mitragynine and 7-hydroxymitragynine are endowed with variable mu, delta, and kappa opioid stimulating properties (with 7-hydroxymitragynine having a more balanced affinity), rhynchophylline, which is a non-competitive NMDA glutamate receptor antagonist, but is present in negligible quantities, and raubasine, which inhibits α_1_-adrenceptors preferentially over α_2_-adrenceptors, while the latter are bound by 7-hydroxymitragynine, while mitragynine counters 5-HT_2A_ receptors. This complexity of neurochemical mechanisms may account for kratom's sedative-analgesic and stimulant effects. It is commonly held that kratom at low doses is stimulant and at higher doses sedative, but no cut-off has been possible to define. Long-term use of kratom may produce physical and psychological effects that are very similar to its withdrawal syndrome, that is, anxiety, irritability, mood, eating, and sleep disorders, other than physical symptoms resembling opiate withdrawal. Kratom's regulatory status varies across countries; in Italy, both mitragynine and the entire tree and its parts are included among regulated substances. We describe the case of a patient who developed anxiety and dysphoric mood and insomnia while using kratom, with these symptoms persisting after withdrawal. He did not respond to a variety of antidepressant combinations and tramadol for various months, and responded after 1 month of clomipramine. Well-being persisted after discontinuing tramadol.

## Introduction

The interest of the medical world in *Mitragyna speciosa* Korthals (MsK) dates back to the1950s (1–6). MsK (kratom) was first described by the Dutch colonial botanist Pieter Korthals in 1839 and is indigenous to Thailand, Indonesia, Malaysia, Myanmar, and Papua New Guinea, where it has been used in traditional medicine and religious (7) contexts since at least the 19^th^ Century, as well as a voluptuary substance use (a surrogate to opium) well before Korthals' description (8, 9). In these countries, leaves of MsK are first dried and then chewed or consumed as smoke in long pipes, extract, or powder, or brewed into a tea (10). Mixtures with other substances are also made, thus increasing dangerousness of consumption. Some of them are confectioned into pills (11). Concern over its use was not raised until recently, when it became largely available in Western countries and its toxic potential realized. A first Malaysian report found kratom consumers to develop addiction and psychiatric symptoms (12), while its psychoactive properties were detailed in the late 1980s (13).

MsK alkaloids were quantitatively determined in its leaves after separation by thin-layer chromatography, with ultraviolet spectrophotometry (14), with colorimetry (15), densitometry (16); indoles and oxindoles were identified in the first place (17). Since then, more than 25 significant alkaloids were identified (11). The corynanthe-type indole mitragynine contributes 66% to MsK alkaloids, paynantheine 9%, 7-hydroxymitragynine 2%, and speciociliatine 1%; other alkaloids contribute <1% each (11). However, their contribution varies across locations (18) and products sold across the world might not always contain MsK at all (19). The first whose structure was determined in 1958 was mitraphylline (20), with many other alkaloids following suit (18). The MsK alkaloids may differ in their brain accessibility and crossing of the blood-brain barrier; for example, mitragynine penetrates in the brain significantly more than 7-hydroxymitragynine, at least in the rat (21). However, the latter is held to be responsible for almost all kratom effects on opioid receptors, and despite low content, it is produced by cytochrome P450 (CYP3A4 isoenzyme) conversion from mitragynine (22).

Other biochemically and neurochemically interesting compounds include rhynchophylline derivatives (23, 24), which down-regulate NMDA-mediated responses in animals (25–27), and a yohimbine and mitragynine analog, ajmalicine or raubasine (28), which differently from mitragynine (29), inhibits α_2_-adrenoceptors, although less than α_1_-adrenoceptors (30–32). Of note, kratom alkaloids closely interact with α_2_ adrenoceptors, and mitragynine and 7-hydroxymitragynine bind them (33). Furthermore, mitragynine inhibits the activity of 5-HT_2A_ receptors, although indirectly so (29, 34, 35), as it shows a *K*_i_ > 10 μM for the 5-HT_2A_ receptor (36). It is possible that the interplay between these receptor effects and between MsK alkaloids underpin the different effects of kratom at low vs. high doses.

Although kratom was reportedly used to substitute for opiate addiction and cure it (37), the demonstration of their binding by MsK had to await the discovery of opioid receptors (38, 39). Mitragynine and other kratom alkaloids were shown to be possibly allosteric (40) agonists to opioid receptors (41–44), to possess analgesic properties thanks to their binding to brain μ- and δ-opioid receptors, and to induce ileal and vas deferens distention through the same receptors at peripheral sites (43, 45). These properties were long harnessed by traditional healers in the countries where kratom grows. In South-East Asia kratom is used to alleviate muscle aches, and sometimes to heal wounds and cure worm infections, while some users support they assume it to increase resistance to fatigue and to stimulate sexuality (46). Indeed, mitragynine and kratom alkaloids are likely to be associated with “dependence” signs and symptoms which are less severe than those usually associated to opiates and they may be used to alleviate classical opiate withdrawal (47, 48). This is not surprising, since they act as agonists on opioid mu receptors.

Legislations concerning kratom varies across countries. In Europe it is illegal in Denmark, Finland, Ireland, Latvia, Lithuania, Poland, Romania, and Sweden (49), while in the UK it has been included in the Psychoactive Substances Bill 2015 (50), hence it is illegal since March 2016, being regulated through the Psychoactive Substances Act 2016 (51). In Italy, it became illegal in 2016. In Canada and Australia, kratom is illegal, while in New Zealand it is a regulated substance. In the United States it is forbidden in some States and not in other (49). Many US state legislations are likely to change their attitude toward kratom in the near future. Similarly, Thailand, one of kratom's major producers, which prohibits since 1943 the cultivation of new plants and mandates the abatement of the existing ones, while restricting possession and use and establishing sanctions for quantities superior or inferior to 10 Kg, is on the verge of changing its legislation. In Malaysia, Bhutan and Myanmar kratom is illegal, and in Indonesia it will be banned by 2022 (52). Discrepancies among the various legislations internationally, as well as the increase in the use of internet and globalization have resulted in an increased use of kratom for voluptuary purposes (53) indicating the need for international coordination of scientists and legislators (54). That kratom could induce an opioid-like withdrawal syndrome, therefore it can be included among addictive substances, is shown by the fact that it may be present in neonates exposed to the substance due to their mothers' heavy use during pregnancy (55–61). In fact, the World Anti-Doping Agency placed mitragynine on its Monitoring List since 2014, and 1 year later, four cases of mitragynine use among strength sportsmen were detected (62). Kratom use has been also reported in fitness settings (63).

The effects of the use of kratom are variable and may depend on the cultural and genetic background of the user as well as on differences in product composition. Product conservation and transport factors may also be involved, as are co-administered sedative or multisubstance use. In a US-Thailand comparison, for example, symptoms were more severe and mortality higher in the US sample, with drowsiness, irritability-agitation and tachycardia being the most common in order of increasing frequency (64). Kratom may be used according to users' taste and adjusted according to the desired effects, with low doses producing stimulant and activating effects and high doses sedative and tranquilizing effects (65–67), although these dose-related effects were not confirmed in a recent study and was unrelated to the amount and duration of kratom use (68). Many people, especially in South-East Asia, get to use kratom after being addicted to opioids and in the attempt to quit; others are prompted to use kratom due to its anxiolytic and mood enhancing effects (69–71). It is expected that upon discontinuing, rebound mood and anxiety symptoms emerge. In regular users, withdrawal symptoms may occur which are more intense in long-time users or after stopping heavy use, and involve usually moderate anxiety and depression (72), as well as aching and disordered sleep (73). However, kratom withdrawal syndromes are usually mild and transient (72–74), similar to but milder than those of opiate withdrawal (74, 75), but may be complicated in some users (54).

We here report the case of an adult man who used kratom and developed withdrawal symptoms while trying to quit. He did well on clompipramine just 1 month after initiation and, 9 months later, is currently symptom-free.

## Case Report

A 44-year-old man, married to a 44-year-old, currently pregnant woman, with a 5-year-old son, a graduate in economy and employed as a researcher at a University, sought help at a community psychiatric service for symptoms of kratom withdrawal and elevated anxiety.

The patient was collaborative at interview, appropriately dressed and well-oriented in time and space; he showed free-floating and somatic anxiety, with tachycardia, profuse sweating, psychomotor agitation, insomnia, dysphoric mood, and emotional lability. His thoughts were focused on anxious experiences and hopelessness. He reported being treated during the last few months with various benzodiazepines and selective serotonin reuptake inhibitors (SSRIs), first paroxetine 40 mg/Day and then sertraline 200 mg/day, to which he associated cognitive-behavioral therapy, with no clear benefit.

The patient had experienced two important major depressive episodes coinciding with stressful life events, which he overcame through the use of SSRIs and long-term psychotherapy. When he was young, he had engaged in polysubstance use, while in his adult life he first used cannabis and alcohol, but later turned to benzodiazepines, alcohol, and kratom, which he obtained through dark internet sites. The patient has been vague as to when and how he started consumption, and also very unclear regarding dosing. His internet-related kratom sources varied, so we are not in a position to determine the purity of the samples he received. During the last 10 months preceding the visit, he had scheduled daily kratom infusions, but had discontinued quite sharply during the last 2 months. The patient used to continue drinking the infusion until he reached the desired effect. Having realized in the last 2 months he was becoming severely dependent, he decided to quit kratom and to no longer seek it on the internet.

Urinary drug testing was positive for benzodiazepines. Blood chemistry showed no abnormal values. However, kratom could not be quantified due to the unavailability of routine laboratory tests. The electrogram (ECG) showed no abnormalities, with a QTc of 385 ms and a heart rate of 60 beats/min. We established treatment with pregabalin 25 mg b.i.d., gradually tapering off sertraline and substituting it with 150 mg/day bupropion, taken in the morning, and 300 mg controlled-release trazodone, administered in the evening. His next visit was scheduled after 2 weeks.

During the second visit, his clinical conditions were unchanged. The patient was restless, anxious, agitated, insomniac, dysphoric, with frequent cry spells and unstructured ideation of self-harm. He craved for benzodiazepines and alcohol and often abused them. Bupropion was increased to 300 mg/day and pregabalin, 75 mg b.i.d. was initiated.

During his third visit, after further 15 days, the above clinical picture persisted. The patient reported to be able to relax, but observed no symptom improvement. We agreed to add 50 mg/day tramadol in the evening. He noted since the first days of tramadol addition a mild reduction in craving and restlessness, with disappearance of self-harm ideas, while anxiety, which the patient reported as paralyzing, dysphoric mood, cry spells, and avolition remained unchanged. The patient asked for a medical certificate to abstain from work, since he considered teaching at the University a complex and stressful activity. We agreed to increase tramadol to 100 mg b.i.d., gradually introducing clomipramine to a target dose of 75 mg/day, while gradually discontinuing bupropion.

Three months after the first visit and about 1 month after introducing clomipramine, the clinical picture was on the way to resolution; the patient himself asked to discontinue tramadol. Free-floating and somatic anxiety had subsided and craving for all substances, including alcohol, benzodiazepines, and kratom, was significantly attenuated, while mood was stable and in the normal range (euthymic).

In the following months, given the clear clinical improvement and the remission of withdrawal symptoms, it was possible to gradually discontinue both pregabalin and trazodone.

Currently, the clinical picture is stable; the patient continues on clomipramine 75 mg/day and about 9 months after its introduction reports to have resumed normal life.

The patient signed free informed consent for the publication of his case and all treatments received.

## Discussion

In this report we presented the case of an adult Italian man in his forties, who deliberately used kratom to soothe his anxiety symptoms. The patient was well-educated and upper socioeconomic class. He had started kratom after engaging in multisubstance use and psychotherapy, while completing steps toward reaching a high social status. He used the internet to obtain kratom, but had no available supplies when he came to our attention, so we could not analyse any kratom specimen he used. After trying several therapeutic strategies, including pharmacotherapy, he was unable to resolve his anxiety symptoms, either during kratom use or during abstinence, and was switched to low-dose clomipramine eventually discontinuing all other psychotherapeutic drugs; 1 month after initiating clomipramine, his symptoms had resolved and so were his anxiety symptoms that had originated psychiatric visits.

There have been several case reports of kratom use toxicity and withdrawal in literature, but clomipramine treatment had not been reported to date. Cases vary in severity and symptom presentation. One of the first described cases of mitragynine toxicity was of severe seizures and come occurring in a 64-year-old man, that resolved soon with symptomatic treatment, that is, intubation to preserve airway integrity (76). In another case, seizures occurred when a 43-year-old man tried to self-treat his opiate dependence with a kratom-modafinil combination (69); the case resolved with few kratom-related withdrawal symptoms. A further case presenting with seizures occurred in a 18-year old man and treated with antiepileptic drugs. Magnetic resonance imaging showed bilateral alterations in the striatum, cerebral peduncles, and subthalamic nuclei in this chronic kratom user, indicating possible permanent effects of kratom in brain structure (77). Finally, a 27-year-old nab with history of anxiety, attention-deficit/hyperactivity disorder, substance use disorders (benzodiazepines and opioids) developed seizures while using kratom and opioids and recovered with anti-anxiety agents (78). Another 36-year-old man was unresponsive to external stimuli and near comatose, did not respond to naloxone and was treated with respiratory support and symptomatic management (79). Another kratom overdose case occurred in a 38-year-old woman who resented with respiratory depression at the emergency department and resolved with naloxone (80). A 33-year-old male polysubstance user exhibited cardiovascular shock features and high procalcitonin levels promptly treated with vasopressors (81). An otherwise healthy 35-year-old man suffered a cardiac arrest after using kratom alone and was found with small brain infarcts, but recovered spontaneously (82). Finally, a 62-year-old woman who used kratom for the first time to soothe traumatic pain presented at the emergency room with intractable vomiting and nausea that responded to ondansetron, promethazine, and famotidine (83).

Cases of kratom-related deaths are usually linked to simultaneous assumption of kratom with other drugs, as in the above described case. Initial death reports regarded associations, but more recent cases show that people who only take kratom are at risk. One case of death of a 20-year-old man occurred with propylhexedrine and kratom; the latter was not determined to have caused the death, which has been associated to accidental propylhexedrine (84). Nine cases of death occurring in one year were described in Sweden in 2011 with the simultaneous intake of mitragynine and *O*-desmethyltramadol. Decedents' age ranged 22–35; seven were men and two were women (85). The authors concluded that mitragynine-related herbal mixes are not so safe as per internet propaganda. Another death case in which mitragynine was involved, but the death was attributed to quetiapine overdose, has been described in a 27-year-old man succumbing to hyperthermia associated with seizures. One case of a 17-year adolescent male who was trying to quit opioid use by self-medicating with kratom, points to kratom being occasionally toxic; the boy was found dead with pulmonary congestion and oedema, as well as urinary bladder distension, which are typical of opiate intoxication (86). The case was labeled as “probable kratom toxicity.” Another case of kratom intoxication-related death was found to be associated with high blood amounts of mitragynine and 7-hydroxymitragynine and unremarkable pathological finding at autopsy in a middle-aged man with psychiatric history and illicit drug use disorder (87). Another report of death related to kratom use was one of a 24-year man with opiate and alcohol use disorders, who was found dead with high peripheral alkaloid concentrations, pulmonary oedema and congestion, and urinary retention, compatible with opioid intoxication; the patient was using several psychiatric medications that were found at therapeutic blood levels (88). Further two cases of young men could not be attributed to the documented kratom use, despite high mitragynine levels in femoral blood (89). One of the patients had attempted suicide just after taking kratom with prescription drugs, while the other took a mix of drugs. Another fatality was due to 3-methoxyphencyclidine, and mitragynine was just one of many other substances the 58-year-old man had taken (90). An emergency case presenting with cardiorespiratory arrest could not be rescued despite the use of intralipid, that nevertheless improved somehow the conditions of a 26-year-old man, but proved ineffective in avoiding exitus, attributed to cardiorespiratory failure and hypoxic brain damage (91). A Canadian 56-year-old woman with chronic obstructive pulmonary disease, after skipping her medication and consuming kratom purchased from Indonesia, died due to respiratory failure (92). Her mitragynine levels in the femoral vein were found to be substantial but sublethal (under the reporting laboratory's threshold of fatality, which was 0.21 mg/L). Another case of multiple drug use ensuing in death has been related to mitragynine due to the very high doses found in inferior cava blood of a 33-year-old man (93). In general, fatality case studies suffer from heterogeneity in kratom alkaloid detection methods and sites.

Cases of chronic kratom used followed by withdrawal symptoms have been reported to resolve with gabapentin in a 26-year-old woman and gabapentin and clonidine in a 27-year-old man (94). A case similar to ours has been described in a 44-year-old man with a history of alcohol use and anxiety; gradually tapered-off dihydrocodeine and lofexidine were followed by rapid withdrawal symptom resolution (95). In our case, the psychiatric symptoms of our patient were more prominent and stubborn, and briefly trialed clonidine in the past had sorted no effect. Hence the need for something more specific for anxiety disorders. Cases of kratom withdrawal in a 47-year-old woman (96) and in a 24-year-old man with an autism spectrum disorder (97) have been treated with clonidine and hydroxyzine, similarly to ordinary opiate withdrawal syndromes. The latter case and four other cases of kratom withdrawal were treated successfully with buprenorphine-naloxone maintenance (98–100). A further withdrawal from combined kratom-tilidine addiction has been successfully treated with retarded morphine (101). Finally, a recent paper reported an unusual presentation of obsessive-compulsive disorder-like syndrome during kratom withdrawal that responded to lorazepam (102). We did not use treatments aimed at treating patient's kratom withdrawal, since the syndrome was mild despite being obstinate, but rather focused on the anxiety disorder, which usually responds to antidepressants. By treating our patient's background psychological symptoms, we were successful in reducing withdrawal symptomatology.

Kratom use has been often linked to liver toxicity. Kratom has been associated with biliary cholangitis and cholestasis in several cases (67, 103–110) and with one case of hepatomegaly (111), but also with acute hepatitis (112). Mitragynine inhibits hepatic and intestinal cytochrome P450 3A activities (113) and hepatic microsomal CYP2D6 (114), thus increasing blood levels of other concomitantly administered drugs that are metabolized by these isoenzymes, that is, most psychiatric drugs. This may expose to further hepatotoxicity (115, 116). Our patient did not develop liver abnormalities during his kratom use period, despite the fact he was concurrently using alcohol. We did not perform kratom quantification analyses in our patient throughout the treatment period. This was because the patient refused to provide organic specimens or leaves for forensic analyses. There are reliable methods for detecting mitragynine and its derivatives in the urine (117) and in plant and extracts (118) for forensic purposes, but these are not currently routine practice. There is need for standardizing methods of kratom alkaloid detection in reported users.

A limitation of the current review is that the supposed benefits-to-risk ratio of kratom use cannot be currently addressed adequately. There is insufficient epidemiological documentation as to the extent of kratom use worldwide and in specific countries (119), so to estimate how many people use it and how many develop unwanted effects. Besides this, risks may increase, as many kratom users have concurrent other substance use (120), and this is difficult to disentangle. The most recent estimates in indicate kratom use in the adult US population is 0.8% for the past year and 1.3% lifetime (120). The debate on epidemiological issues is strong and ongoing, and points to the evergreen “more studies are needed” (121, 122). The advocates of kratom use to ease opioid dependence and harness its effects on strength and endurance while involved in work activities do not publish in scientific literature, but put forward their uncontrolled views and opinions in sites of their own property. Hence, it is an impervious task to try to respond to the question whether kratom use is relatively safe, but it appears it is not (123). Currently, there is not sufficient evidence to recommend changes in kratom regulation, nor to recommend the use of clomipramine in cases of kratom withdrawal.

Our patient showed while withdrawing from kratom mitigated signs and symptoms typical of opiate withdrawal, which were mixed with other psychiatric symptoms presumably linked to his background psychopathology. Knowing that the withdrawal is generally time-limited and mild, we chose to use an anxiety-specific agent, clomipramine, with preference for serotonin transporter over noradrenaline transporter inhibition, which is a tricyclic antidepressant used in anxiety disorders and obsessive-compulsive disorder and has shown good evidence in these disorders. We used it at 75 mg/day, which is on the lower range of clinical effectiveness for these disorders. Mitragynine counters serotonin 5-HT_2A_ receptors (29) and clomipramine down-regulates the same receptors after chronic treatment (124, 125); furthermore, it has pain supressing effects even at low doses through spinal mechanisms (126). Hence, it is possible that some mitragynine withdrawal symptoms were alleviated concomitantly with clomipramine's anxiety relieving effects. However, this is not the most likely mechanism whereby clomipramine reduced our patient's symptomatology. In fact, clomipramine may obviate for the opiate-like mitragynine withdrawal syndrome through interference with opioid receptors, which it was shown to bind (127); chronic, but not subacute clomipramine administration, induced a mu receptor down-regulation in the rat (128). In this case, clomipramine could reduce the quantity of opioid receptors in the need for occupation, as it occurs in opiate withdrawal. However, the response of human opioid receptors to chronic clomipramine appears to be weak (129). We are unsure about how improvement was obtained, but the timeline appears to match the usual onset of clomipramine antidepressant effects.

## Concluding Remarks

Summarizing the above evidence, we may conclude that kratom may induce addiction, acute toxicity which may be sometimes lethal and, upon discontinuation, it induces a withdrawal syndrome, which may vary in intensity. In many instances that appeared in literature, kratom was regularly used by patients with psychiatric history and/or substance use disorders. Legislations should take very seriously peer-reviewed published evidence and regulate the substance. In parallel, we need to enforce kratom detection methods in consent-providing users for forensic purposes. International drug policies should be coordinated and inform the public about kratom and other novel addictive drugs.

## Data Availability Statement

The original contributions presented in the study are included in the article/supplementary material, further inquiries can be directed to the corresponding author/s.

## Ethics Statement

The patient signed free informed consent for the publication of his case and all treatments received.

## Author Contributions

AV, SP, and SD saw the patient and wrote the first draft. FS, FN, and JC supervised the case and the writing of the manuscript. GK wrote the last draft and performed literature searches. All authors saw and approved the final version of the manuscript.

## Conflict of Interest

The authors declare that the research was conducted in the absence of any commercial or financial relationships that could be construed as a potential conflict of interest.
